# Update and Potential Opportunities in CBP [Cyclic Adenosine Monophosphate (cAMP) Response Element-Binding Protein (CREB)-Binding Protein] Research Using Computational Techniques

**DOI:** 10.1007/s10930-020-09951-8

**Published:** 2021-01-04

**Authors:** Oluwayimika E. Akinsiku, Opeyemi S. Soremekun, Mahmoud E. S. Soliman

**Affiliations:** Molecular Bio-computation and Drug Design Laboratory, School of Health Sciences, University of KwaZulu-Natal, Westville Campus, Durban, 4001 South Africa

**Keywords:** CREB, Molecular dynamic simulation, CREB inhibitors, Bromodomains

## Abstract

CBP [cyclic adenosine monophosphate (cAMP) response element-binding protein (CREB)-binding protein] is one of the most researched proteins for its therapeutic function. Several studies have identified its vast functions and interactions with other transcription factors to initiate cellular signals of survival. In cancer and other diseases such as Alzheimer’s, Rubinstein-taybi syndrome, and inflammatory diseases, CBP has been implicated and hence an attractive target in drug design and development. In this review, we explore the various computational techniques that have been used in CBP research, furthermore we identified computational gaps that could be explored to facilitate the development of highly therapeutic CBP inhibitors.

## Introduction

**Fig. 1:**
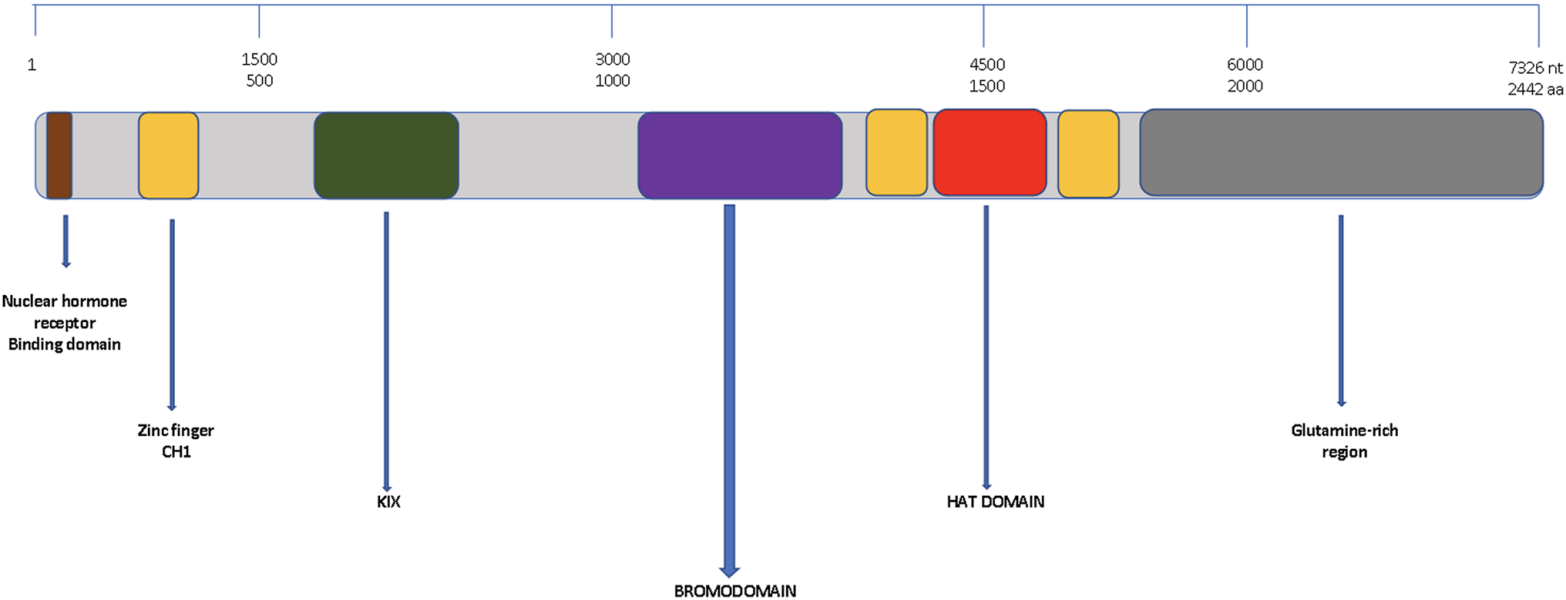
CBP and its interacting domains

The CREB (cyclic adenosine monophosphate (cAMP) response element-binding protein) Binding Protein (CBP), is a protein encoded by the CREBBP gene. CBP is a bromodomain-containing protein which emphasises its functionality in identifying acetylated lysine in histone proteins while also acting as effectors in signal associated with acetylation [1]. This class of protein has been reported to play a significant role in many biological and physiological processes, including transcription, differentiation, and apoptosis, whose activity is regulated by phosphorylation [1]. It’s unique structure is made up of domains that catalyses transcription process initiated in cell growth, gene expression and differentiation as shown in Fig. 1. The histone acetyltransferase (HATs) domain, also part of the CREB binding protein is necessary for protein–protein interactions, histone and non-histone alike such as NCOA3 and FOXO1. In 1993, p300, a Switch/Sucrose Non-Fermentable (SWI/SNF) complexes binding protein family was identified. It was discovered to share similarity with CBP in terms of its bromodomain, HATs domain and the cysteine-histidine region [2]. Despite this similarities, they both cannot be used interchangeably. Ryan et al., researched for their differences and identified that their selectivity for lysine within the histones is the major reason for their differences [3]. Although, CBP are coactivators of transcription, they do not interact with the promoter element. Instead, they are mobilized to promoters by protein–protein interaction [1, 4, 5]. The CREB binding protein has a binding domain called the KIX (kinase inducible domain) or the CREB binding domain [4]. This CREB (cAMP-response element-binding protein) unit within CBP controls the rate of transcription when phosphorylated at Ser-133 residues through protein kinase A which triggers the transcription activity of CBP [6]. The transactivation domain of CREB is bipartite, which consist of a glutamine-rich constructive activated site called Q2 and kinase-inducible domain (KID), and are directly in response to gene expression [7]. Despite the phosphorylation interaction between cAMP-dependent PKA and CREB, it is still unknown whether phosphorylation on the amino acid Ser-133 elicit CREB-CBP complexation. The mechanism of interaction is still not precise, either direct or allosteric [6].

### Bromodomain: What About It?

**Fig. 2:**
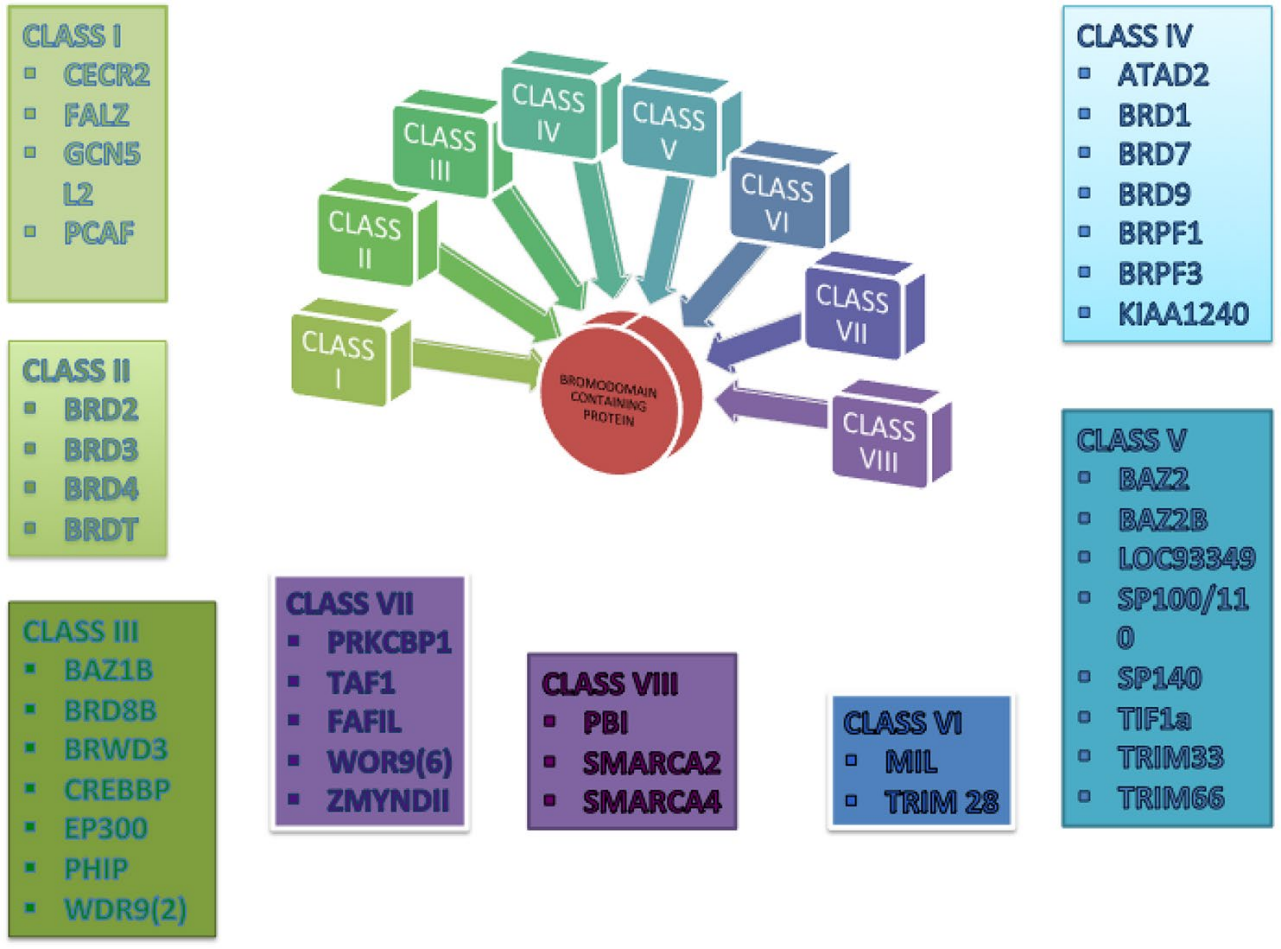
Classification of the different classes of BET Proteins (prepared by the author)

Wetlaufer defined protein domains as stable units of protein structure, possessing structural and evolutionary functions that fold autonomously [1]. Bromodomains (BRDs) are parts of a given protein sequence (approximately 110 amino acids) that recognizes lysine acetylation of N-terminal histones during gene transcription [1]. They are responsible for histone acetylation, chromatin remoulding, and transcription activation [8]. John Wetlaufer Tamkun first proposed the discovery of bromodomain-proteins while studying the drosophila gene Brahma [9]. PCAF, histone acetyltransferase (HATs) KAT2B was the first 3-dimensional structure of BRD to be solved using NMR spectroscopy in 1999 [8]. Bromodomains are also called histone code readers [10, 11]. Of all the proteins in the human proteome, there are 61 BRDs, and based on their structure–function relationship, they are grouped into eight subfamilies [1]. These BRDs all have four α-helices linked by loops of different lengths (a, b, c and z) with which it interacts with acetylated lysine residues. These helices are coiled up in a left-handed α-helical fold. Between helix b and c and helix z and a, there are two loops forming a hydrophobic pocket [12]. The differences shown in the binding of bromodomains are due to the differences in sequence beyond the residues bound directly with acetyl-lysine binding [12–14] Although each protein is specific with its structure yet 48 of the more than 61 BRDs contain the asparagine residue at the acetyl-lysine binding site (KAc recognition position) while the remaining 13 have a tyrosine, threonine or an aspartate in the same position. The latter is called atypical BRDs [15]. There are eight subgroups of the BRDs classified in accordance to their amino acid sequence similarities as seen in Fig. 2 above (*Classification of the different classes of BET Proteins*). They are the BET family, histone acetyltransferases HATs (GCN5, PCAF), methyltransferases (MLL, ASH1L), ATP-dependent chromatin-remodelling complexes (BAZ1B), helicases (MARCA), nuclear-scaffolding proteins (PB1) and transcriptional coactivators (TRIM/TIF1, TAFs) transcriptional mediators (TAF1) [13]. Specific sub-groups have gained more attention compared to others; this is partly due to the development of inhibitors targeting BRDs. Of all the BRDs, the BET (bromodomain and extra-terminal family) BRDs (BRD2, BRD3, BRD4, and BRDT) are most researched and has over 206 PBD structures available today [13].

## CREB-Binding Protein (CBP)

**Fig. 3:**
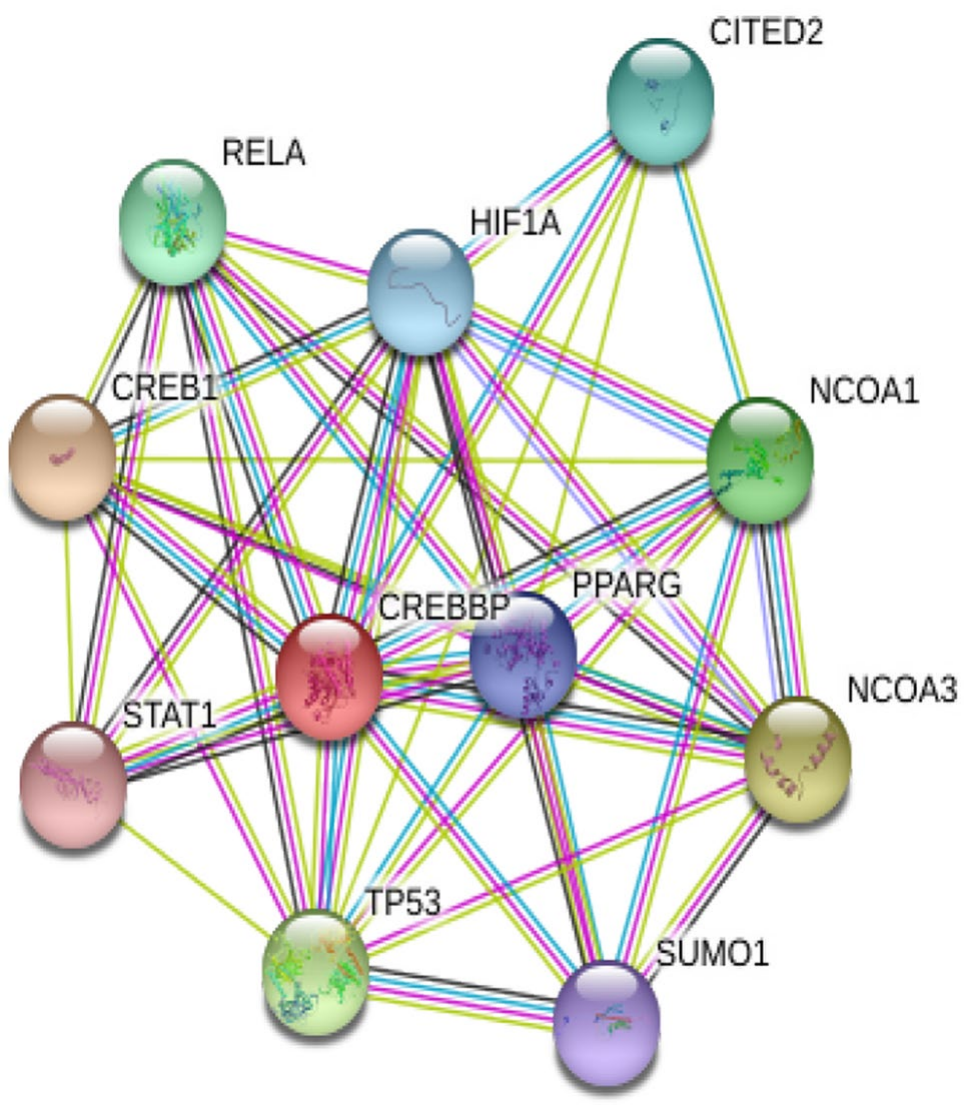
A database report from STRING showing the functional interactions of CREBBP with other proteins

CBP is a nuclear protein of Mr 265 K that bounds to phosphorylated cAMP-regulated transcription factor CREB, this fusion allows CBP to function as protein kinase A-regulated transcriptional activator [16, 17]. Both CBP and p300, its analogous, shares a few functional domains in common which constitute their similarities: (1) they are BRDs which are commonly found in human HATs; (2) they both have domains of the three cysteine-histidine namely CH1, CH2, and CH3; (3) they both have the KIX domain; and (4) an ADA2-homology domain [18]. Despite the broad structural similarities, Ho Man Chan and Nicholas Thangue attest to the unique characteristics of CBP and p300 [19]. Also, both CBP and p300 are phosphorylated at the different amino acid sites; CBP is phosphorylated at serine 436, an amino acid absent in p300 [20] which is absent in the latter. On a quick database check on STRING, CBP is shown to interact with the following proteins as shown in the Fig. 3. Such proteins include NCOA3, TP53, NCOA1, RELA, CITED2, HIIF1A, PPARG, SUMO1 and STAT1. Meanwhile, Intact database reports a more detailed interactions of 790 binary proteins. In 1996, p300 and CBP were reported to function as histone acetyltransferases (HATs). CBP especially was discovered to possess intrinsic histone acetyltransferase activity even though it lacked conserved motifs found in regular acetyltransferases. With this property in view, it is only direct to suggest that it modulates cell cycle progression. It is demonstrated to acetylate nucleosomes associated with PCAF [21, 22]. CBP has been shown to play a vital role in gene expression. A study reported CBP as a HAT capable of acetylating nuclear factor-4 (HNF-4) of liver cells at lysine residues inside the nuclear localization sequence [23]. CBP continues to be of great interest in the development and design of drugs CBP plays an extensively role at the molecular level, such as, cellular growth, histone acetylation, and transcription of some factors amidst other unique functions. For example, CBP brings about the assembly of multi-protein complexes, which serves as molecular scaffolds [19]. CBP, along with other transcription factors, are known to regulate the overall process involved in the cell, including gene transcription [24]. It is essential to the point that in transforming viral proteins such as E1A from adenovirus, CBP is a prerequisite target [25]. Also, another review suggests that CBP proteins are targets for adenovirus E1A oncoprotein indicating its vital role in cell cycle regulation [5]. Observations by Ait-Si-Ali et al., reported that HAT is involved in the cell cycle by the phosphorylation of CBP by cyclin-E-CDK2 in the C-terminal region of the protein hence stimulating HAT activity [26]. Moreover, the results indicated that E1A activates the CBP HAT enzyme on the binding, which then results in a conformational change in its domain, leading to an increased catalytic activity. CBP interacts with viral oncoproteins such as p53 to cause loss of cell growth or growth suppression. p53 interacts with a carboxyl-terminal region of CBP and activate genes involved in DNA damage and block cellular differentiation such as p21, murine double minute (MDM-2), BAX and cyclin G [27, 28].

## CREB-Binding Protein (CBP) and the Onset of Diseases

CBP’s function in cancer was first identified in the translocation of chromosome t(12;22) q(13;12). Studies have shown that CBP is involved in all stages of tumour development, in addition to its being a proto-oncogene. A statistic of patients with prostate cancer, lung cancer, acute leukaemia, and breast cancer showed overexpression and over activation of CBP [29]. Also, the inhibition of cell proliferation and induction of apoptosis was observed in the downregulation of CBP, which suggests that it as a prospective target for cancer therapy [30]. Although the involvement of CBP in cancer development is not explicit yet, CBP directly controls genes critical to cell progression, growth, and metastasis. CBP has also been identified in the development of embryos and cancer [21]. In Alzheimer’s disease, CBP activator (CREB1), together with CBP, enhances memory formation and learning [31]. However, in certain circumstances, increase in CREB1 function can also alter cognitive performance. A publication by Tang et al., aimed to search the function of CREB1 in the onset of Alzheimer’s diseases (AD) [31]. The result implicated CREB1 and CBP as the culprit in the pathophysiology of Alzheimer’s disease (AD), yet further research could be done on a much larger population to confirm these observations [31]. A research was conducted to analyse the function of CBP in inflammatory diseases. It turned out that few studies have been reported in line with rheumatoid arthritis (RA) synovial fibroblasts (SF). Results showed that the inhibition of CBP has an anti-inflammatory effect, while p300 showed both pro and anti-inflammatory functions [32].

### Various Attempt to Target CBP

**Fig. 4:**
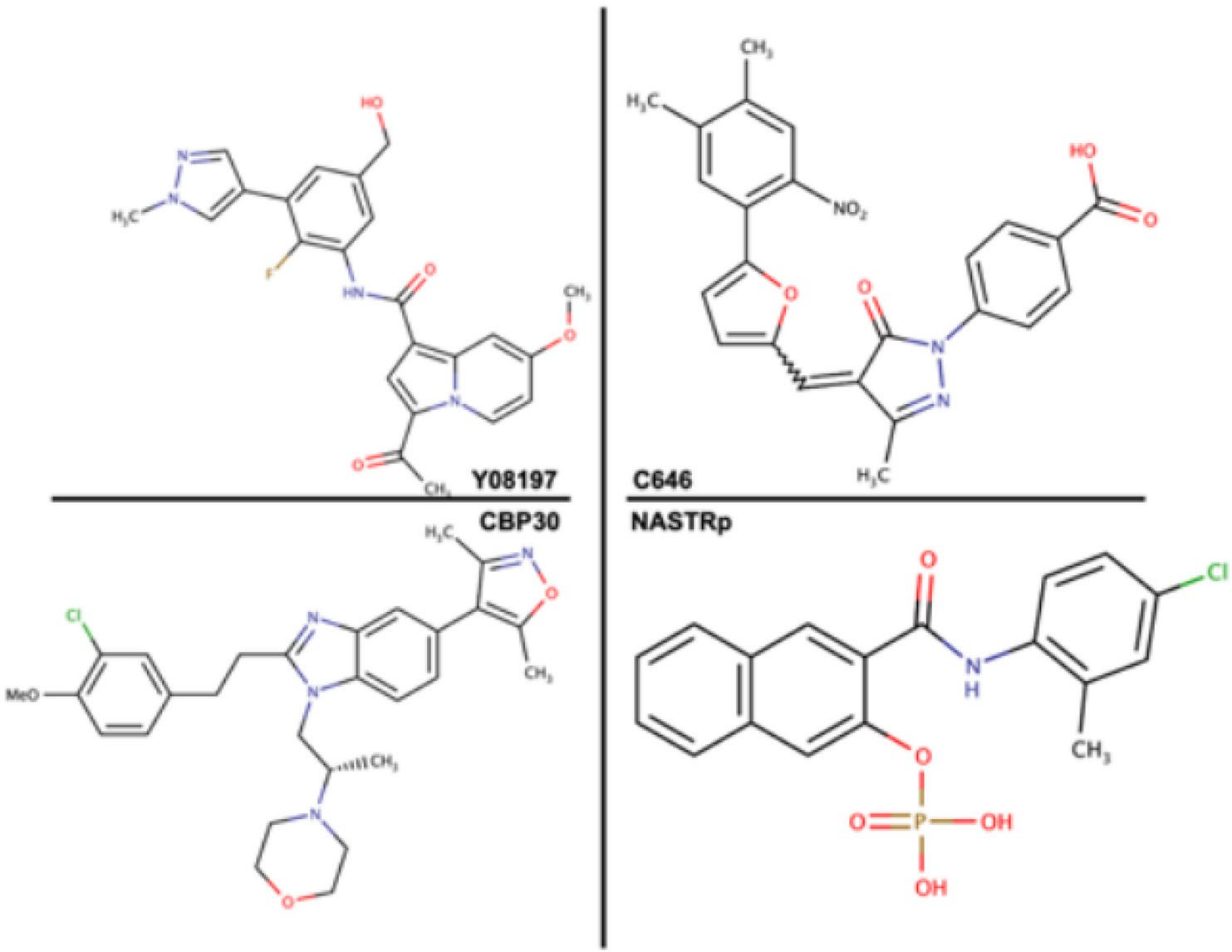
2D Structures of CREB inhibitors (as prepared by the author)

Recently, Hammitzsch et al., developed a CBP inhibitor (CBP 30) to block Th17 responses in human autoimmune diseases. Th17 has been proven to be very vital to various human autoimmune diseases. In the above research, the inhibitor blocked the bromodomain of the coactivator CBP, showing remarkable results [33]. Although the inhibitor was tested with about 43 bromodomain binding protein, excellent result that far exceeds even the known JQ1 (a BET inhibitor) was observed. In castration-resistant prostate cancer (CRPC), an advanced prostate cancer, CBP, and its homolog p300 are highly expressed. Given this, various therapy is aimed towards blocking the activity of CBP. In a recent study, YO8197, a selective inhibitor of CBP bromodomain was explored in terms of its antitumor activity against prostate cancer cell lines in vitro [34] of which further in silico studies by akinsiku et al., proved the mechanistic and selective targeting of Y08197 at the bromodomain site. Asp 116 was identified as the culprit responsible for the selective targeting [35]. Another CBP inhibitor, C646 has been investigated against neuroepithelial cell proliferation [36]. This study by Bai et al., further justified the abnormality in NE-4C cells of CBP in high glucose. With the administration of C646 to the diabetic induced mouse, the results indicated that the levels of acetylation were reduced. Conclusively, it was evident that C646 could effectively impede the increase of histone H4 acetylation and neuro-epithelial cell proliferation [36, 37]. Statistics reports that 1% of pregnant women are affected by diabetes and might have congenital heart disorder and neural tube defects (NTDs) in the child born [38]. Figure 4 shows 2D-structure of CREB-BP inhibitors and Table 1 explains in detail the drugs experimentally designed to target CBP as discussed.

**Table 1:** A table showing the various drugs experimentally designed to target CBP for different diseases with necessary details

S/N	Drugs	Experiments	Diseases targeted	Results	Ref
01	CBP 30	In vitro	Human autoimmune diseases	Inhibited IL-17A secretion via Th cells from healthy donors	[33]
02	Y08197	In vitro	Castration resistance prostrate cancer	Affected the downstream signalling transduction, inhibiting expression of AR-related genes	[34]
03	C646	In vitro In vivo	Neuroepithelial Cell proliferation	Rescued increased H4k5/k8/k12/k16 acetylation levels	[36]
04	NASTRp (Naphthol AS-TR phosphate)	In vitro	Lung adenocarcinoma	Inhibited oncogenic cells via cell cycle arrest and also initiated downregulations of Atg5-12 and Atg7	[40]
05	Compound DC_ CP20	In silico	Human leukaemia	Inhibited the proliferation of human leukaemia MV4-11 cells and downregulated the expression of c-Myc in the cells	[41]
06	NEO2734	In vitro In vivo	Prostate cancer	Inhibition of cell growth with a significant effect compared to a combination of JQ1 and CPI-637	[43]
07	Nicur	In silico	Gastrointestinal epithelial cells	Blocked CBP HAT activity and down regulates p53 activation upon cellular responses	[66]

Recent research proved that NASTRp is effective in inhibiting cancer cells via cell arrest [39]. Since mutant KRAS drives the activation of CAMP responsive element-binding (CREB), it is only appropriate to devise an inhibitor that can effectively do such through RAF/MEK/ERK signalling pathway inducing apoptosis in cancer cells [40]. Compound DC_CP20, a new CBP BRD inhibitor, discovered through a time-resolved fluorescence energy transfer (TR-FRET)-based high throughput screening of about 20 000 libraries of compounds [41]. An IC50 of 744.3 nM was demonstrated when bound with the acetylated lysine of CBP BRD. Moreover, with the aid of molecular docking, the binding affinity was further juxtaposed, being bound tightly in the inner Kac-binding pocket competitively. The compound proves an inhibitory property to human leukaemia MV4-11 cells at cellular levels. These promising results pose a further study in the development of drug therapies for CBP-related cancers [42]. Studies have shown the frequent occurrence of SPOP (speckle-type POZ protein), a mutated gene in primary prostate cancer (Pca) in about 10 to 15% range [39]. A study by Yuqian Yan et al., identified an unknown mutation called Q165P at the cliff of the SPOP math domain [43]. The effect of this mutation is that it halts the dimerization of SPOP, and consequently substrate degradation. Furthermore, unlike F133V, the former is highly sensitive to the known BET inhibitor, JQ1. In vivo and in vitro experiments carried out revealed a novel BET and CBP inhibitor, NEO2734, is effective against the JQ1-resistant SPOP hotspot mutant, which could proceed further to clinical trials for effective anti-cancer therapy against SPOP-mutated PCa patients [43].

### Computer-Aided Techniques in Studies of CREB-Binding Protein

Over the years, traditional strategies used in drug development and design pipeline have been complemented with computational software and methods. These tools include; pharmacophore modelling, molecular docking, virtual screening, molecular dynamics (MD) simulation, Quantitative Structure–Activity Relationship (QSAR), and homology modelling. Computer-aided drug design techniques have been effective over the years in finding new drugs from genomic and proteomic initiatives. These new techniques have effectively reduced cost and increased drug discovery. Molecular docking have been adopted over the years and involve ligand-receptor orientation to find the best conformation of fitness that would trigger a biological response. Some popular docking programs are FlexX [44], GOLD [45], AutoDock [46], GLIDE [47], DOCK [48, 49], HEX SERVER [50], Surflex [51], Patchdock [52] among others.

The importance of molecular dynamics (MD) simulation cannot be overemphasized, especially with its coherent contribution to the interplay between computational and experimental techniques. These step-by-step techniques effectively reveal the dynamic behaviour of the proteins at timescales intervals, the stability of the protein structure, and the ligand’s binding interactions. Other properties such as conductivity, dipolar moment, density, thermodynamic parameters, entropies, amidst others. are observed [53–56]. MD simulation programs include CHARMM [57], NAMD [58], GROMACS [59], AMBER [60], among others. We searched some published papers with an emphasis on the computational methods that have been adopted in CREB research. A paper by Woo Lee published in 2015 reports the anti-cancer properties of Naphthol AS-TR phosphate (NASTRp), a novel CREB-CBP Complex inhibitor with many functions. Among all compounds, NASTRp showed the best effect, especially in biological assays. In this research, computational tools were employed in conducting a database search of compounds with possible chemical properties. Using the DBslnfilter, compounds were screened under properties such as no 3D coordinates, mixtures, isotopes, Molecular Weight < 100, or Molecular Weight > 500, metals. In this structural database are approximately 600,000 compounds that also contain about 50 chemical databases [61]. These compounds are usually downloaded in the SDF file format [62], followed by a database search command investigation on each compound to identify any two-dimensional similarity. Compounds were screened using PubChem, after which a four-processor MIPS R16000 Silicon Graphics Tezro was used to conduct modelling calculations. The results were then combined into 3-D SLNs. All Compounds not containing carboxylates, phosphates, and sulfonamides were eliminated using the hit list manager. The PDB ID: IKDX represents the KIX domain coordinates. This result from taking the average of the NMR structures with the phoenix Elbow [63] the resultant produces the KIX and NASTRp coordinates. The docking calculations were obtained using HEX 6.3 [64]. The result indicated that out of the calculations of the top ten docking scores, NASTRp was shown to have the best binding score. Although molecular simulation wasn’t carried out to accompany the experiment yet, the results indicate NASTRp as a potential anti-cancer drug. Researchers over the years have shown great interest in investigating CBP as a potential drug target, as shown in some few works demonstrated in advanced MD simulations. Md simulation was conducted to decipher the mechanism of the selective inhibitor CBP30 against its target CBP/p300 bromodomain. It was discovered that the specific residue for CBP, Arg1173/1137, was accountable for the selective binding to CBP30 through hydrogen bond interactions and cation–π. In order to prove the result, four (4) system was set up; the apo-CBP, CBP-CBP30 complex, apo-p300, p300-CBP30 complex. Observing the interactions, CBP30 ring B formed a contact collision with the Arg1173 side-chain of Apo-CBP, meanwhile forming a favourable cation–π between the holo-CBP. For as long as 93% simulation time, the cation–π interaction was preserved. CBP, both contact and cation–π interaction reflected in apo-p300 and CBP 30, yet another H-bond is seen between CBP30 O3 and Arg1137 NH1 atoms of holo-p300. With these results, a greater understanding is known of the mechanism of CBP30 against BET and non-BET bromodomains [65]. Vincek et al., 2018, identified a CBP inhibitor, NiCur, and further proved its ability to block the activity of CBP HAT as well as the regulation of p53 activation upon genotoxic stress downstream via computational studies [66]. NiCur was docked using Autodock-4 [46] into the active site of the CBP HAT and poses generated showed its binding affinity. A group of researchers reviewed the result of docking fragment-based high throughput ligands in rigid binding targets of the N-terminal BRD of BRD4 and CREBBP bromodomain [65]. In silico screening was aided with the newly developed procedure based on fragment for high throughput docking of large libraries of compounds. These compounds are called anchor-based library tailoring (ALTA) [46]. Of over 2 million compounds decomposed using the DAIM program [67], approximately 97 fragments with either hydrogen bond donor or acceptor and a ring were parameterized using MATCH [68]. These compounds, with the use of SEED [69, 70], were docked into two structures of CBP. Only 4000 fragments survived the double filtering stage, of which the best compounds continued the docking process in the ALTA procedure using AutoDock Vina [46]. Poses were minimized with CHARMM. Remarkably, only 20 compounds emerged the best in terms of their interaction with the asparagine residue in the binding target. Since the aim of the experiment involved its definition of the stability of the interaction, 100 ns molecular simulation was carried out with each docked pose. It was reported that the ethylbenzene derivatives showed greater efficiency and binding selectivity compared to other CBP bromodomain inhibitors (SGC-CBP30) [71] and I-CBP112 [72] reported by others.

## Conclusion

This study proves the progression of CREB-BP from concept to computational research. Its unique properties have been evaluated through times and have been a significant target, especially in cancer drug development. Various inhibitors have been identified, and the investigation continues to emerge in its progression to being drugs for diseases. Having looked into examples of studies in which MD simulation and docking were adopted, it is quite evident that more progress is likely to be seen in this continuous study.
